# VATICAN (Ventilator-Associated Tracheobronchitis Initiative to Conduct Antibiotic Evaluation): protocol for a multicenter randomized open-label trial of watchful waiting *versus* antimicrobial therapy for ventilator-associated tracheobronchitis

**DOI:** 10.62675/2965-2774.20240029-en

**Published:** 2024-08-05

**Authors:** IMPACTO-MR, VATICAN Trial Investigators and BRICNet

**Affiliations:** 1 Hospital Sírio-Libanês São Paulo SP Brazil Hospital Sírio-Libanês - São Paulo (SP), Brazil.; 2 Brazilian Research in Intensive Care Network São Paulo SP Brazil Brazilian Research in Intensive Care Network (BRICNet) - São Paulo (SP), Brazil.; 3 Hospital das Clínicas Faculdade de Medicina Universidade de São Paulo São Paulo SP Brazil Trauma and Acute Care Surgery Intensive Care Unit, Hospital das Clínicas, Faculdade de Medicina, Universidade de São Paulo - São Paulo (SP), Brazil.; 4 Hospital Tricentenário Olinda PE Brazil Hospital Tricentenário - Olinda (PE), Brazil.; 5 Hospital Israelita Albert Einstein São Paulo SP Brazil Hospital Israelita Albert Einstein - São Paulo (SP), Brazil.; 6 A Beneficência Portuguesa de São Paulo São Paulo SP Brazil BP - A Beneficência Portuguesa de São Paulo - São Paulo (SP), Brazil.; 7 Hospital Moinhos de Vento Porto Alegre RS Brazil Hospital Moinhos de Vento - Porto Alegre (RS), Brazil.; 8 Hospital Alemão Oswaldo Cruz São Paulo SP Brazil Hospital Alemão Oswaldo Cruz - São Paulo (SP), Brazil.; 9 Research Institute Hospital do Coração São Paulo SP Brazil Research Institute, HCor-Hospital do Coração - São Paulo (SP), Brazil.; 10 Universidade Federal de São Paulo São Paulo SP Brazil Universidade Federal de São Paulo - São Paulo (SP), Brazil.

**Keywords:** Bronchitis, Pneumonia ventilator-associated, Respiratory tract infections, Antibacterial agents, Critical care, Critical care outcomes, Respiration, artificial, Ventilators, mechanical

## Abstract

**Background:**

Ventilator-associated tracheobronchitis is a common condition among invasively ventilated patients in intensive care units, for which the best treatment strategy is currently unknown. We designed the VATICAN (Ventilator-Associated Tracheobronchitis Initiative to Conduct Antibiotic Evaluation) trial to assess whether a watchful waiting antibiotic treatment strategy is noninferior to routine antibiotic treatment for ventilator-associated tracheobronchitis regarding days free of mechanical ventilation.

**Methods:**

VATICAN is a randomized, controlled, open-label, multicenter noninferiority trial. Patients with suspected ventilator-associated tracheobronchitis without evidence of ventilator-associated pneumonia or hemodynamic instability due to probable infection will be assigned to either a watchful waiting strategy, without antimicrobial administration for ventilator-associated tracheobronchitis and prescription of antimicrobials only in cases of ventilator-associated pneumonia, sepsis or septic shock, or another infectious diagnosis, or to a routine antimicrobial treatment strategy for seven days. The primary outcome will be mechanical ventilation-free days at 28 days, and a key secondary outcome will be ventilator-associated pneumonia-free survival. Through an intention-to-treat framework with a per-protocol sensitivity analysis, the primary outcome analysis will address noninferiority with a 20% margin, which translates to a 1.5 difference in ventilator-free days. Other analyses will follow a superiority analysis framework.

**Conclusion:**

The VATICAN trial will follow all national and international ethical standards. We aim to publish the trial in a high-visibility general journal and present it at critical care and infectious disease conferences for dissemination. These results will likely be immediately applicable to the bedside upon trial completion and will provide information with a low risk of bias for guideline development.

## INTRODUCTION

The use of invasive mechanical ventilation (IMV) for organ support is common in intensive care units (ICUs), with an estimated prevalence ranging from 36 to 89%.^(1-3)^ Despite being a potential life-saving procedure, IMV is associated with complications, such as ventilator-associated pneumonia (VAP) and ventilator-associated tracheobronchitis (VAT).^(4,5)^ Although the incidence of these complications has decreased in recent decades due to improvements in preventive measures and care processes,^(6)^ it is estimated that the incidence of VAT is still 11% in patients receiving IMV.^(7)^ Patients with VAT have longer durations of mechanical ventilation, ICU stays, and hospital stays.^(7,8)^ Estimates suggest that approximately 12% of cases of VAT progress to VAP,^(7)^ suggesting that these two entities may be on a *continuum*.^(9)^ However, the relationship between VAT and increased mortality is unclear.^(7)^

Although there are many studies evaluating strategies for VAT prevention,^(10)^ there are limited data from clinical trials to determine whether VAT warrants antibiotic treatment, and only one ongoing clinical trial, which is still unpublished.^(11)^ Observational data have shown a possible benefit of antibiotic treatment in reducing VAT progression to VAP^(7,12)^ and a possible mortality benefit,^(13)^ but a meta-analysis showed no benefit of antibiotic treatment on clinical outcomes.^(10)^ Furthermore, the Infectious Diseases Society of America (IDSA) guidelines^(5)^ recommend against the routine treatment of VAT with antibiotics (weak recommendation, low quality of evidence). An online survey comprising 288 ICUs from 16 countries^(14)^ revealed considerable heterogeneity in the use of antibiotics for VAT treatment, with 42% of respondents reporting antibiotic use for all VAT patients, while 26% did not use it at all; the remainder used antimicrobials only under specific circumstances, such as in the presence of cardiovascular dysfunction. Hence, an apparent discrepancy is observed between clinical practice and current guidelines. These limitations of the current evidence are recognized by the heterogeneity in VAT treatment in clinical practice and reinforce the need for further studies in the field.^(5,6,15-17)^

The VATICAN (Ventilator-Associated Tracheobronchitis Initiative to Conduct Antibiotic Evaluation) trial is designed to address this gap by assessing whether a watchful waiting antibiotic treatment strategy is noninferior to routine antibiotic treatment for VAT regarding mechanical ventilation-free days (VFDs) among invasively ventilated patients with a clinical diagnosis of VAT. We hypothesize that the watchful waiting strategy will lead to less antimicrobial consumption with no impact on mortality or mechanical ventilation duration at the possible expense of an increased risk of VAP.

## METHODS

We used the recommendations for Interventional Trials (SPIRIT) guidelines for this report.^(18)^The final report will follow the Consolidated Standards of Reporting Trials (CONSORT) statement and its extension to noninferiority trials.^(19)^ The trial was registered with ClinicalTrials.gov (NCT05266066) before inclusion of the first patient.

### Design

The VATICAN is an investigator-initiated, multicenter, randomized, controlled, open-label, noninferiority clinical trial conducted to compare whether a watchful waiting antibiotic treatment strategy is noninferior to antibiotic treatment for seven days for VAT treatment.

The trial will be conducted in up to 50 Brazilian ICUs. The trial is partially nested in the IMPACTO MR platform,^(20)^ a research platform that collects prospective observational data from more than 50 ICUs in Brazil, with the VATICAN trial also allowing for the inclusion of ICUs outside the IMPACTO MR platform.

### Trial population

**Figure 1:**
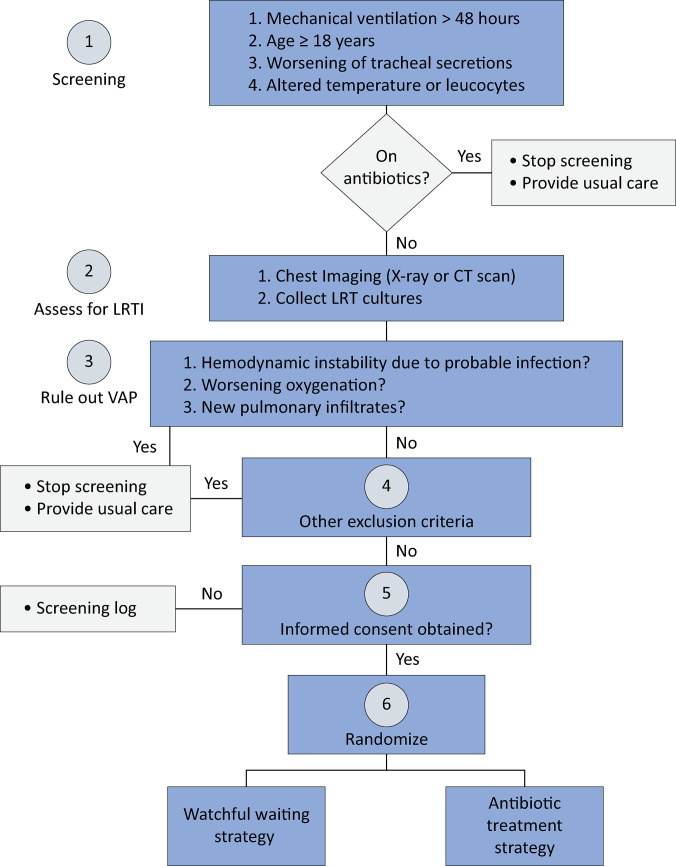
Screening of trial participants and triage into the trial up to randomization. LRTI - lower respiratory tract infection; CT - computed tomography; LRT - lower respiratory tract; VAP - ventilator-associated pneumonia.

The target population of the VATICAN trial is patients with suspected VAT who do not have septic shock or clinical evidence of VAP. Patients who are mechanically ventilated for more than 48 hours at each participant site will be assessed daily by site investigators for eligibility. Mechanically ventilated patients will be screened during the first three weeks of mechanical ventilation, i.e., not on prolonged mechanical ventilation. We aim to include patients who met the following clinical criteria for VAT: (1) had at least one event of leukocytosis, leukopenia, left shift, fever, or hypothermia, and (2) met at least one criterion for worsening tracheal secretions. The main exclusion criteria were patients with hemodynamic instability due to probable infection, highly suspected VAP (either because of an evident new pulmonary infiltrate or persistently worsening gas exchange), who were currently on antimicrobial agents or who had another indication for antimicrobial treatment. These exclusion criteria ensure that no patients randomized in the trial will have a Clinical Pulmonary Infection Score (CPIS) ≥ 7, which was shown in a recent study to be very specific (0.89) and have a high positive predictive value (0.88) for VAP diagnosis.^(21)^Figure 1 presents the screening and eligibility evaluation for recruitment into the trial. The detailed inclusion and exclusion criteria are described in table 1.

**Table 1:** Inclusion and exclusion criteria of the VATICAN trial

**Inclusion criteria**
1. Adult patients (age ≥ 18 years old)
2. Admitted to ICU
3. Invasive mechanical ventilation for at least 48 hours
4. Presence of clinical criteria for VAP, defined as: a. Temperature > 38°C or < 36°C **OR** leucocyte > 12,000/mL or < 4,000/mL or > 10% immature forms **AND** b. New onset of purulent tracheal secretions **OR** change in secretion characteristics **OR** increase in secretions volume **OR** increased suctioning requirements
5. Lower respiratory tract microbiological sample under analysis or collection on the day of trial triage
6. Chest imaging (chest X-ray or computed tomography) on the day of trial triage
**Exclusion criteria**
1. Pregnancy or active lactation
2. Current use or indication of use of systemic antibiotics for any reasons
3. Hemodynamic instability, defined as new-onset hypotension unresponsive to fluid boluses or increase in vasopressor dose > 0.1mcg/kg/minute of norepinephrine or equivalent in the previous 6 hours
4. Worsening gas exchange, defined as an increase in the oxygen-inspired fraction ≥ 20% or an increase in PEEP ≥ 3cmH_2_O after a period of stability of ≥ 2 days
5. Prolonged mechanical ventilation, defined as an invasive mechanical ventilation duration ≥ 21 days
6. A new pulmonary opacity on chest image suggestive of new infectious infiltrates
7. Previous pulmonary disease that hampers the radiographic interpretation for VAP diagnosis
8. Previous diagnosis of VAP during hospitalization
9. Neutropenia (absolute neutrophil count < 1,000/mL)
10. Severe known immunosuppression
11. Tracheostomy on the day of trial triage
12. Prior inclusion in the trial in the last 30 days
13. Expected withholding or withdrawing of life support within 7 days
14. Patients unlikely to survive > 48 hours
15. Consent refusal to participate in the trial

ICU - intensive care unit; VAP - ventilator-associated pneumonia; PEEP - positive end-expiratory pressure.

### Randomization, allocation concealment, and blinding

Patients will be randomized in a 1:1 ratio to each treatment arm (Watchful Waiting Group *versus* Antibiotic Treatment Group for 7 days). Randomization lists will be generated by an independent statistician using R software^(22)^ in random block sizes to preserve allocation concealment and will be stratified by center and by suspected diagnosis of viral pneumonia. Randomization will be performed using a central online randomization system (RedCap) that is available 24 hours a day.

This is an open-label trial in which both patients and investigators are not blinded to the interventions, given the nature of the trial and the need to follow local treatment regimens based on local microbiology. The adjudicators and trial statisticians will be blinded to the treatment assignment.

### Trial treatments

**Figure 2:**
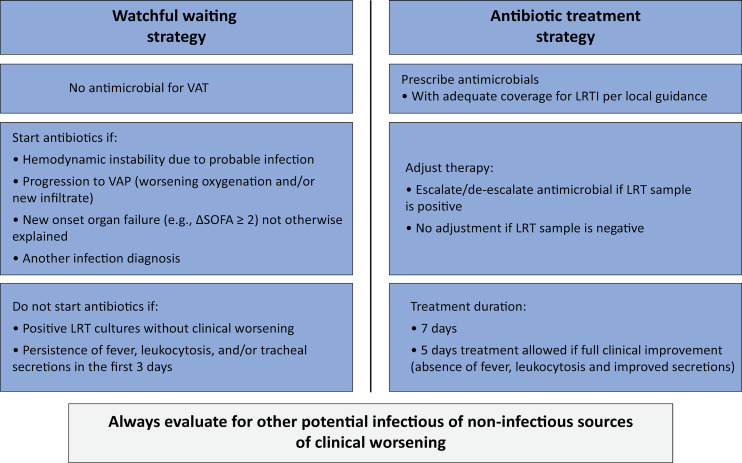
Trial procedure description during the 7-day treatment protocol window. VAT - ventilator-associated tracheobronchitis; LRTI - lower respiratory tract infection VAP - ventilator-associated pneumonia; SOFA - Sequential Organ Failure Assessment; LRT - lower respiratory tract.

Figure 2 presents the main elements of the trial protocol. In the Watchful Waiting Group, participants will not receive antimicrobials for VAT. Antimicrobials can be used, however, at the clinician’s discretion when other non-VAT-related indications ensue, such as progression to VAP, hemodynamic worsening or shock, newly confirmed or highly probable infection by the treating physician, unexplained new or worsened organ dysfunction or any other validated clinical indication for antimicrobial utilization. Antimicrobial initiation is not advised if lower respiratory tract cultures are positive and the patient does not develop other treatment indications.

In the Antibiotic Treatment Group, participants received antimicrobials for seven days for the treatment of VAT. Five days of treatment is allowed for patients with complete clinical improvement, defined by the absence of fever, leukocytosis and improvement in tracheal secretions. Adjustments to the empirical antimicrobial treatment will be made after microbiological results are available, as appropriate. The antimicrobial choice will align with the recommended empirical antimicrobial choice for ICU-acquired lower respiratory tract infections based on the microbiota of each unit. We provide guideline-recommended antimicrobial choices in centers where an empirical regimen is not suggested. Antimicrobials for non-VAT-related indications will also be allowed in the antibiotic treatment group.

Each ICU enrolling patients in the trial is encouraged to follow the best practice guidelines and their institutional protocol for the care of critically ill patients,^(23)^ including a VAP prevention protocol. Nevertheless, the VATICAN trial will not directly influence best practices at each site. Inhaled antimicrobial agents will not be allowed during the trial. More information on the trial’s procedures is available in the Supplementary Material.

### Protocol deviations

As the trial is open-label, protocol deviations may occur. Furthermore, due to its noninferiority nature and the need for per-protocol analyses, protocol deviations will be followed closely and evaluated by the coordinating center in accordance with predefined criteria.

In the Antibiotic Therapy Group, protocol deviations will be considered if the patient does not receive antibiotics within 24 hours after randomization or if the patient receives less than seven days of antibiotic treatment, except for patients eligible for a shorter treatment duration (patients who have complete clinical improvement) who should receive at least five days of antibiotic treatment.

In the Watchful Waiting Group, antimicrobial initiation after randomization within the 7-day treatment protocol window will be considered a protocol deviation in the absence of another clear indication for antibiotic treatment initiation, as described above.

### Trial outcomes

The primary outcome is VFD at 28 days. Ventilator-free days are defined as being free of IMV for at least 48 hours (successful extubation).^(24)^ If the patient is reintubated within 48 hours of extubation, this interval will be treated as zero VFDs. If reintubation is needed after 48 hours, this period will be counted as the VFDs. Patients who undergo a tracheostomy will be considered free of mechanical ventilation once they tolerate continuous nebulization (with or without oxygen), allowing for short periods (< 1 hour/day) of positive pressure ventilation. Patients who are discharged from the hospital alive before 28 days will be considered alive and free of mechanical ventilation at 28 days. Nonsurvivors up to 28 days will be considered to have zero VFDs.

The secondary outcomes are VAP-free survival at 28 days (key secondary outcome), all-cause mortality at 28 days, VAP incidence at 14 and 28 days, number of ICU-free days at 28 days, number of antibiotic-free days at 28 days, change in the Sequential Organ Failure Assessment (SOFA) score between randomization and 7 days and cumulative incidence of microbiologically isolated MDR bacteria at 28 days. Secondary safety outcomes are the incidence of culture-positive hospital-acquired infections at 28 days and severe adverse events at 28 days.

The VAP outcome will be adjudicated by at least two adjudicators who are blinded to the trial’s intervention and who have experience in the care of critically ill mechanically ventilated patients. If discrepancies arise, they will be resolved by consensus between both adjudicators. If no consensus is reached, a third adjudicator will be responsible for making a final decision on the diagnosis of VAP.

Detailed information on the outcomes and trial definitions is available in the Supplementary Material.

### Data collection

Patient data will be collected through an online case report form (e-CRF) using REDCap,^(25,26)^ developed by the data management team of Hospital Sírio-Libanês. Access to the e-CRFs will be password-protected and supported by the trial data management team.

Demographic and baseline data, including the Simplified Acute Physiology Score (SAPS) 3, laboratory data, data on physiological variables, previous antibiotic use, and use of steroids upon randomization, among others, were collected for all participants. Daily data on antibiotic utilization, microbiological cultures, and ventilatory support were collected for up to 28 days. Additional information on the data collection is available in the Supplementary Material.

### Data monitoring

A designed centralized data manager will perform the data monitoring for quality and completeness. Source data will also be routinely audited online or on-site according to a predefined procedure. During data collection, fields were explicitly designed to allow high-quality data to be collected using ranges and validation criteria. Further details on the data monitoring are described in the Supplementary Material.

### Adverse events

This is a trial in critically ill patients, and we acknowledge that, due to their clinical status, there is an inherent high risk of death and of several laboratory abnormalities, vital sign abnormalities, and symptoms directly related to the underlying illness severity, as well as usual treatment practices in ICUs. Therefore, these abnormalities will not necessarily be considered adverse events unless there is a suspicion or confirmation that they are related to the trial interventions.^(27)^ All severe adverse events potentially causally related to the trial intervention will be reported and investigated per GCP guidance.

### Sample size

The sample size calculation was based on the demonstration of noninferiority of the watchful waiting strategy compared to the use of antibiotics for 7 days strategy. A sample size of 295 patients per group (total of 590 patients), with a one-sided t test for noninferiority (with a significance level of 0.05), has 80% power to reject the null hypothesis that the number of VFDs at 28 days in the Watchful Waiting Group is inferior to the number of VFDs at 28 days in the Antibiotic Treatment Group for 7 days by a margin of 20% and a coefficient of variation of 1.38,^(7)^ considering a potential loss to follow-up or dropouts of 10%.

The choice of the 20% noninferiority margin was motivated by what was considered acceptable by the Steering Committee from a clinical perspective. This 20% noninferiority margin translates into a 1.5 VFD difference at 28 days, considering the mean VFD of 7.69 days and the standard deviation (10.67) in patients with VAT from a prior publication.^(7)^

### Interim analysis

We plan to conduct interim analyses after the inclusion of 1/3 (196 subjects) and 2/3 (392 subjects) of the estimated sample, which will be performed by an independent Data and Safety Monitoring Board. The goal of this analysis is to stop the trial early for safety reasons if clearly superior outcomes are observed in the antibiotic treatment group regarding the primary outcome (VFDs), which will be determined using the Haybittle-Peto boundary^(28)^ (p < 0.001) in each interim analysis, without adjustments of the p value for group sequential analyses. There was no specific a priori planning to terminate the trial for futility or efficacy.

### Statistical analysis

The main analysis will follow the intent-to-treat framework, where the null hypothesis is that the number of VFDs up to 28 days in the Watchful Waiting Group is less than the number of VFDs in the Antibiotic Treatment Group for 7 days by a margin of 20%. If the upper bound of the 95% confidence interval for inferiority of the Watchful Waiting Group is < 20%, we will reject the null hypothesis of inferiority. To estimate the effect of treatment on the primary outcome, as well as in the interim analyses, an appropriate statistical model adjusted for stratification variables will be used for this type of outcome according to the variable behavior: either a generalized additive model for location scale and shape with zero-inflated beta distribution or nonparametric tests with the calculation of measures of effect and confidence intervals using resampling techniques (bootstrap) can be used. The effect size will be estimated according to the model best fitted to the data, with a 1-sided 95% confidence interval (95%CI) for noninferiority. If the noninferiority hypothesis is satisfied, superiority for the primary outcome will be tested considering a 95%CI using a hierarchical closed testing procedure. A primary sensitivity analysis of this noninferiority analysis will be a per-protocol analysis. Although per-protocol analyses are usually the primary analysis framework recommended for noninferiority trials, the intention-to-treat analysis framework may lead to more conservative estimates in antimicrobial trials.^(29)^ A p value of 0.05 will be considered to indicate statistical significance in the final analysis.

We also performed preplanned sensitivity analyses of the primary outcome, including only patients with microbiologically confirmed VAT, and we analyzed the randomization groups as an instrumental variable. Preplanned subgroup analyses will include the following: patients with or without a diagnosis of coronavirus disease 2019 (COVID-19) or other viral pneumonia, age ≤ 60 or > 60 years old, diagnosis of trauma on admission, acute brain injury, invasive mechanical ventilation duration of ≤ 5 or > 5 days at the time of randomization and effective vs. ineffective empirical antimicrobial treatment for isolated pathogens.

For the outcomes including VAP as a secondary outcome, we will perform sensitivity analyses based on the definition of VAP, including the more specific and more sensitive definition of VAP (including possible and probable cases of VAP). These definitions are presented in the Supplemental Material.

All secondary analyses will follow the usual superiority framework for analysis. Further details on the statistical model for the primary outcome, secondary outcomes, sensitivity and subgroup analyses, and data presentation will be described in detail in the statistical analysis plan to be published before database closure, in accordance with international recommendations for reporting randomized clinical trials.

### Ethics and dissemination

Participants will not be capable of providing consent due to their clinical condition (use of IMV). Therefore, consent will be obtained from their next-of-kin and/or legal representative. Given the timeliness of the intervention, participants can be randomized, and consent can be obtained within 48 hours after their inclusion in the trial. The trial is designed according to the guidelines for good clinical practice and follows the principles of the *Documento das Américas*, and it was approved by the *Hospital Sírio-Libanês* Ethical Committee (CAAEE 48749421.0.1001.5461) and by the research ethical committees of all participant sites.

Confidentiality will be ensured throughout the entire process, from data collection to data management. Only unidentifiable data will be shared.

The trial will be submitted for publication after completion, irrespective of its findings. We aim to publish the results in a high-visibility general journal and present it at critical care and infectious disease conferences for dissemination. The data generated from this trial will be made available upon request to the trial’s Steering Committee with a scientifically sound research proposal.

## DISCUSSION

The VATICAN is a large multicenter, randomized clinical trial evaluating whether VAT, in the absence of signs of septic shock or high-risk criteria for VAP, should be treated. Given its open-label nature, it should be viewed as a trial of antibiotic treatment strategies, not of antibiotic treatment itself. These results are likely to inform future guidelines about this condition.

When designing the VATICAN trial, the investigators examined essential issues that merit discussion. The most fundamental issue was designing a trial that enhanced the recruitment of centers on both ends of the equipoise for this trial, i.e., both elements for those who feel strong about routinely treating VAT and those who feel strongly about not treating VAT except in exceptional circumstances.

The VATICAN was initially considered an efficacy superiority trial of the 7-day treatment arm, given the current guideline recommendations not to treat VAT routinely. However, after discussion within the trial Steering Committee, the feedback obtained from likely participating centers, and considering the currently published evidence that treatment is commonly introduced, we decided that noninferiority hypothesis testing would be more likely to provide evidence beyond a reasonable doubt, especially for physicians who feel strongly about treating VAT. Robust evidence is essential if a clinical trial ultimately aims to change clinical practice. Since the noninferiority hypothesis led to a larger sample size, if any of the arms perform better than others, an efficacy analysis can be conducted using standard frequentist approaches and acceptable type I error rates.

Blinding and the choice of antimicrobial agent within each site are also concerns. Given the varying microbiology of lower respiratory tract infections in Brazilian ICUs,^(30)^ it was unlikely that a single antimicrobial treatment strategy would be appropriate for all participating sites. Furthermore, such a tactic would not represent the sites’ clinical practice and would ultimately hamper trial generalizability. Therefore, we decided that the best design strategy would be an unblinded design where the choice of antimicrobial agent is left to the trial site according to local antimicrobial resistance patterns. This strategy poses additional challenges in trial implementation but increases uptake of the intervention and center recruitment. In contrast, how antimicrobials are prescribed will not be monitored due to the pragmatic nature of the delivery of the intervention, although this may be a potential limitation.

Participant recruitment into was another concern during the trial design. Unpublished Brazilian data suggest that a vast majority of invasively ventilated ICU patients undergo antimicrobial treatment for a long duration of their stay. When recruiting sites, we reinforce the commitment of individual sites to the rational use of antimicrobials, which could be viewed as a positive unintended consequence of the trial. As recruitment rolls out, this is a critical planned part of site visits to optimize recruitment.

Another pitfall in the trial is the adherence to the interventions, given its unblinded nature and how strongly individual clinicians may feel regarding antimicrobial therapy, where clinicians are usually biased toward using antimicrobial therapy more than necessary for many reasons.^(31)^ This issue will be specifically monitored during the trial roll-out by evaluating the separation between treatment groups in accordance with antibiotic treatment during the first seven days after randomization. To reduce the potential biases in the final analysis, we reinforced the adjudication of protocol deviations with clear criteria, such as those described above. Treatment duration is another design choice of our trial. We chose 7 days of treatment in the routine treatment arm to allow physiological separation from the Watchful Waiting Group, but we do not consider it a protocol deviation if the treatment is shortened to 5 days in patients with full clinical improvement. Shorter (3 days) treatment durations have been suggested, but these are not currently standard practice in Brazilian ICUs.

Finally, the choice of the primary outcome was another important issue in trial design. While the incidence of VAP may be seen as an interesting outcome for the VATICAN trial, recent trials assessing VAP as a primary outcome have not shown benefits in other clinical outcomes when VAP is prevented,^(32,33)^ suggesting that VAP may be best viewed as a surrogate endpoint that is not necessarily related to other clinical outcomes. Therefore, we chose VFDs as our primary outcome, which would balance the benefits and harms of the intervention in a single, more patient-centered, composite outcome. Additionally, other secondary outcomes, such as antibiotic-free days and ICU-free days, will provide additional elements for assessment of the benefits and harms of treatment regimens.

### Perspective

As we wrote the draft for this protocol, more than 180 patients were included in the trial, and 32 sites have been trained and recruited since June 2022. With up to 50 active sites, we expect the trial to be completed by 2026. However, the above-described pitfalls and barriers to recruitment may lead to slower recruitment rates, which will be addressed by the trial Management Committee.

## CONCLUSION

The VATICAN trial represents a considerable effort to evaluate a long-standing research question that has not yet been addressed by adequately powered multicenter randomized trials. The results are likely to be immediately applicable to the bedside upon trial completion and will provide information with a low risk of bias for guideline development.

## SUPPLEMENTARY MATERIAL

VATICAN (Ventilator-Associated Tracheobronchitis Initiative to Conduct Antibiotic Evaluation): protocol for a multicenter randomized open-label trial of watchful waiting *versus* antimicrobial therapy for ventilator-associated tracheobronchitis


