# Supplementary material for: VATICAN (Ventilator-Associated Tracheobronchitis Initiative to Conduct Antibiotic Evaluation): protocol for a multicenter randomized open-label trial of watchful waiting versus antimicrobial therapy for ventilator-associated tracheobronchitis
Source: Crit Care Sci. 2024 Aug 5;36:e20240029en. doi: 10.62675/2965-2774.20240029-en (PMC11321716; doi:10.62675/2965-2774.20240029-en)
Supplement: Supplementary file 1 [file 2965-2774-ccsci-36-e20240029en-suppl.pdf]

Critical Care Science
https://doi.org/10.62675/2965-2774.20240029-en

SUPPLEMENTARY MATERIAL

VATICAN (Ventilator-Associated Tracheobronchitis Initiative to Conduct Antibiotic Evaluation): protocol for a multicenter randomized open-label trial of watchful waiting *versus* antimicrobial therapy for ventilator-associated tracheobronchitis

Bruno Martins Tomazini[1,2], Bruno Adler Maccagnan Pinheiro Besen[1,2], Camila Dietrich[1], Ana Paula Rossi Gandara[1], Debora Patrícia Silva[1], Carla Cristina Gomes Pinheiro[1], Mariane Nascimento Luz[1], Renata Rodrigues de Mattos[1], Luiz Fernando Lima Reis[1], Roberta Muriel Longo Roepke[3], Carlos Sérgio Luna Gomes Duarte[4], Antônio Paulo Nassar Júnior[2,5], Viviane Cordeiro Veiga[2,6], Beatriz Arns[7], Giovanna Marssola Nascimento[8], Adriano José Pereira[2,5], Alexandre Biasi Cavalcanti[2,9], Flávia Ribeiro Machado[2,10], Luciano Cesar Pontes Azevedo[2,5], for the IMPACTO-MR, VATICAN Trial Investigators and BRICNet

SUMMARY

Additional information on study definitions and outcomes 2
Ventilator-free days definition 2
Ventilator-associated pneumonia-free survival definition 2
Intensive care unit free days definition 2
Antibiotics-free days at 28 days 2
Ventilator-associated pneumonia diagnosis 2
    Clinical ventilator-associated pneumonia 2
    Microbiological definition of ventilator-associated pneumonia 2
Multidrug resistant microorganism definition 3
Cut-offs for lower respiratory tract culture results 3
Additional information on eligibility and interruption of study participation 3
    Criteria for interruption of study participation 3
Additional information on data collection and monitoring 3
    Data monitoring 3
Additional information on study's procedures 3
Infection adjudication and effectiveness of antimicrobial therapy 3
References 4

This is an open access article under the CC BY license (https://creativecommons.org/licenses/by/4.0/).

ADDITIONAL INFORMATION ON STUDY DEFINITIONS AND OUTCOMES

Ventilator-free days definition

Ventilator-free days (VFD) is defined as being free of invasive mechanical ventilation for at least 48 hours (successful extubation).[1] If the patient is reintubated within 48 hours of extubation, this time period will be considered as zero VFDs. If reintubated after 48 hours, this period will be counted as VFDs. Patients who undergo a tracheostomy will be considered free of mechanical ventilation once they tolerate continuous nebulization (with or without oxygen), allowing for short periods (< 1 hour) of positive pressure ventilation. Patients who are discharged alive before 28 days will be considered alive and free of mechanical ventilation at 28 days. Non-survivors up to 28 days will be considered as having zero VFDs.

Ventilator-associated pneumonia-free survival definition

Ventilator-associated pneumonia (VAP)-free survival is defined as the time between the patient's entry into the study and the diagnostic event of VAP or death. All patients will be censored within 28 days or upon hospital discharge, whichever comes first.

Intensive care unit-free days definition

Intensive care unit (ICU)-free days is defined as being alive and free of the ICU for at least 24 hours. Patients who are discharged alive before 28 days will be considered alive and free of ICU at day 28. Non-survivors at 28 days will be considered as having zero ICU-free days.

Antibiotics-free days at 28 days

Antibiotics-free days will be defined as being alive and free of antibiotics for at least 24 hours. Patients who are discharged alive before 28 days will be considered alive and free of antibiotics at day 28. Non-survivors at 28 days will be considered as having zero antibiotics-free days.

Ventilator-associated pneumonia diagnosis

The diagnosis of VAP will follow the Agência Nacional de Vigilância Sanitária's (ANVISA) definitions.[2] In this trial, we will use both clinical and microbiological definitions of VAP according to the following criteria:

Clinical ventilator-associated pneumonia

1. Patient under mechanical ventilation for at least 48 hours or mechanical ventilation has been removed in the last day, **AND**
2. New, persistent, or progressive chest imaging infiltrate, opacity or cavitation, **AND**
3. At least one of the following: increase in body temperature >38.0°C **OR** leucocyte count > 12000/mL or < 4000/mL, **AND**
4. Presence of:
    a. New onset of purulent tracheal secretions, or change in the respiratory secretion characteristics, or increase in the amount of respiratory secretions, or increased need of tracheal aspiration, **AND**
    b. Worsening of gas exchange.

Microbiological definition of ventilator-associated pneumonia

1. Fulfillment of all clinical VAP criteria, **AND**
2. At least one of the results below:
    a. Positive blood cultures, without other infectious source, **OR**
    b. Positive pleural fluid cultures, **OR**
    c. Positive quantitative culture of a lower respiratory tract specimen obtained with a minimal potential for contamination (bronchoalveolar lavage, protected brush or endotracheal aspirate), **OR**
    d. On bronchoalveolar lavage bacterioscopy, finding of ≥ 5% of leucocytes and macrophages with microorganisms, **OR**
    e. Positive culture from lung tissue, **OR**
    f. Histopathological exam with evidence of pneumonia, **OR**
    g. Viruses, *Bordetella*, *Legionella*, *Chlamydophila* or *Mycoplasma* identified from a culture of a lower respiratory tract specimen or lung tissue or identified through a microbiological test done for diagnostic or treatment reasons, **OR**
    h. 4-fold increase in IgG values serology for a pathogen (e.g. *influenza*, *Chlamydophila*), **OR**
    i. 4-fold increase in IgG values serology for *Legionella pneumophila* serogroup I titrated to ≥ 1:128 in the acute phase or convalescence by indirect immunofluorescence assay, **OR**
    j. Urinary antigen detection of *Legionella pneumophila* serogroup I

MULTIDRUG RESISTANT MICROORGANISM DEFINITION

The operational definition of multidrug resistant microorganisms is described below

Microorganism	Resistance profile
Acinetobacter baumannii	Resistant to carbapenems and/or polymyxins
Pseudomonas aeruginosa	Resistant to carbapenems and/or polymyxins
Enterobacteriaceae	Resistant to carbapenems and/or polymyxins (for *Enterobacteriaceae* naturally sensitive to polymyxins)
Enterococcus faecium	Resistant to vancomycin
Staphylococcus aureus	Resistant to methicillin/oxacillin
Coagulase-negative Staphylococcus	Resistant to methicillin/oxacillin

CUT-OFFS FOR LOWER RESPIRATORY TRACT CULTURE RESULTS

The cut-offs for lower respiratory tract culture results are described below.

Method	Cut-off
Endotracheal aspirate	$\geq 10^6$ colony-forming units/mL
Bronchoalveolar lavage	$\geq 10^4$ colony-forming units/mL
Protect brush specimen	$\geq 10^3$ colony-forming units/mL
Semi-quantitative culture	Moderate or high growth

ADDITIONAL INFORMATION ON ELIGIBILITY AND INTERRUPTION OF STUDY PARTICIPATION

Criteria for interruption of study participation

The consent withdrawal by the study participant or their surrogate decision maker will imply in interruption of study participation. However, in this case, the subject or surrogate will be asked for consent to use their collected data. If consent is not granted, data will be excluded from analysis.

ADDITIONAL INFORMATION ON DATA COLLECTION AND MONITORING

Research personnel delegated at each participant center will perform the data collection. Additionally, all microbiological cultures results will be uploaded to the electronic case report form (eCRF) for all patients during the first 28 days or until hospital discharge, whichever comes first. Also, if during the study period, new antibiotics are initiated or new infections are suspected, a copy of the last two medical notes will be uploaded. If the suspected infection is pulmonary, radiological images will be uploaded. All files will be deidentified prior to upload. All healthcare professionals participating in the trial will undergo standardized training for data collection through electronic conferences or on-site visits. Coordinating center personnel will be available by 24/7 telephone support, text messages or e-mail.

Data monitoring

All patients included in the trial will be monitored as described in the study's monitoring plan. A monitoring team from the coordinating center will be responsible for all monitoring activities, which will be performed either by on-site or online visits consisted of two main aspects.

1. Documentation monitoring: monitoring of ethical approvals, good clinical practice (GCP) certificates, delegation forms, Institutional Review Board (IRB) reports (including adverse events reporting), informed consent of recruited patients, and training logs.
2. Patient data monitoring: Source data verification in all included patients of all intervention, outcomes, and infection data. This also comprises monitoring of all documentation sent for adjudicators.

ADDITIONAL INFORMATION ON STUDY'S PROCEDURES

Participant institutions will receive an evidence-based guide for antimicrobial utilization considering pathogen sensitivity to probable lower respiratory infections with the aim of homogeneity in treatment choices, which should be based on the bacterial resistance profile of each institution. However, the final choice of antimicrobials to be used either in the antimicrobial for 7 days group or other cases of lower respiratory tract infections that demand antimicrobial utilization will be at the discretion of the treating clinician in charge of the patient.

Participant centers will also be subject to good clinical practice in adequate sample collection for cultures when a suspected infection occurs, once microbiological data is available, escalation or de-escalation of antibiotics is recommended in the antimicrobial for 7 days group.

INFECTION ADJUDICATION AND EFFECTIVENESS OF ANTIMICROBIAL THERAPY

Two adjudicators will be responsible for evaluating all the information concerning infections and effectiveness of

antimicrobial therapy (for VAT and all possible infections that might occur during the study) during the study period. Infection adjudications will be blinded to the intervention group.

Regarding the effectiveness of antimicrobial therapy, each antimicrobial regimen will be classified as:

- Appropriate: when at least one antimicrobial used to treat the infection has in vitro activity against the isolated pathogen and has adequate penetration in the infection site.
- Inappropriate: when the isolated pathogen has in vitro resistance to all antimicrobials used for treating the infection or has inadequate penetration in the infection site.
- Indeterminate: when there is no pathogen isolated for that specific infection or the antibiotics used were not evaluated in the antibiogram.

All possible infections that might occur in both groups are evaluated. Adjudicators use a combination of data inserted on the eCRF, together with copies of medical records, radiology exams, and microbiological cultures results. Additional information might be requested to each site center if needed. For diagnosis of VAP we will follow the ANVISA's definitions as previously described. Other possible infections will be classified as suggested by Klein Klouwenberg et al.:[3]

- Not infected: no clinical, laboratory or microbiological findings that suggest an infection or a definitive alternative explanation for clinical and laboratory findings.
- Possible infection: either all necessary information needed for a precise classification is not present in the medical records and other files evaluated or subtle physiological changes that might suggest inflammation but without microbiological confirmation.
- Probable or confirmed infection: unequivocal evidence of signs of infection, sepsis or septic shock, regardless of microbiological cultures, or infections with microbiological confirmation in sterile sites (confirmed) or non-sterile sites (probable) associated with evidence of infection.

For patients presenting with two or more same infection diagnosis within 14 days, we will only consider the first episode.

REFERENCES

1. Beduneau G, Pham T, Schortgen F, Piquilloud L, Zogheib E, Jonas M, Grelon F, Runge I, Nicolas Terzi, Grangé S, Barberet G, Guitard PG, Frat JP, Constan A, Chretien JM, Mancebo J, Mercat A, Richard JM, Brochard L; WIND (Weaning according to a New Definition) Study Group and the REVA (Réseau Européen de Recherche en Ventilation Artificielle) Network. Epidemiology of weaning outcome according to a new definition. The WIND study. Am J Respir Crit Care Med. 2017;195(6):772-83.
2. Agência Nacional de Vigilância Sanitária (ANVISA). Critérios diagnósticos das infecções relacionadas à assistência à saúde. Brasília (DF): ANVISA; 2021. [Nota Técnica GVIMS/GGTES/ANVISA nº 02/2021].
3. Klein Klouwenberg PM, Ong DS, Bos LD, de Beer FM, van Hooijdonk RT, Huson MA, et al. Interobserver agreement of Centers for Disease Control and Prevention criteria for classifying infections in critically ill patients. Crit Care Med. 2013;41(10):2373-8.
