# Supplementary material for: VATICAN (Ventilator-Associated Tracheobronchitis Initiative to Conduct Antibiotic Evaluation): protocol for a multicenter randomized open-label trial of watchful waiting versus antimicrobial therapy for ventilator-associated tracheobronchitis
Source: Crit Care Sci. 2024 Aug 5;36:e20240029en. doi: 10.62675/2965-2774.20240029-en (PMC11321716; doi:10.62675/2965-2774.20240029-en)
Supplement: Translation

# Translation: VATICAN (*Ventilator-Associated Tracheobronchitis Initiative to Conduct Antibiotic Evaluation*): protocolo para um ensaio multicêntrico aberto e randomizado de espera vigilante *versus* terapia antimicrobiana em traqueobronquite associada ao ventilador

**DOI:** 10.62675/2965-2774.20240029-pt

**Authors:** IMPACTO-MR, VATICAN Trial Investigators and BRICNet

**Affiliations:** 1 Brasil Hospital Sírio-Libanês - São Paulo (SP), Brasil.; 2 Brasil Brazilian Research in Intensive Care Network (BRICNet) - São Paulo (SP), Brasil.; 3 Brasil Unidade de Terapia Intensiva de Cirurgia de Trauma e Cuidados Agudos, Hospital das Clínicas, Faculdade de Medicina, Universidade de São Paulo - São Paulo (SP), Brasil.; 4 Brasil Hospital Tricentenário - Olinda (PE), Brasil.; 5 Brasil Hospital Israelita Albert Einstein - São Paulo (SP), Brasil.; 6 Brasil BP - A Beneficência Portuguesa de São Paulo - São Paulo (SP), Brasil.; 7 Brasil Hospital Moinhos de Vento - Porto Alegre (RS), Brasil.; 8 Brasil Hospital Alemão Oswaldo Cruz - São Paulo (SP), Brasil.; 9 Brasil Instituto de Pesquisa, HCor-Hospital do Coração - São Paulo (SP), Brasil.; 10 Brasil Universidade Federal de São Paulo - São Paulo (SP), Brasil.

**Keywords:** Bronquite, Pneumonia associada à ventilação mecânica, Infecções do trato respiratório, Antibacterianos, Cuidados críticos, Resultados de cuidados críticos, Respiração artificial, Ventiladores mecânicos

## Abstract

**Contexto:**

A traqueobronquite associada ao ventilador é uma condição comum entre pacientes ventilados invasivamente em unidades de terapia intensiva, para a qual se desconhece atualmente a melhor estratégia de tratamento. Desenhamos o estudo VATICAN (*Ventilator-Associated Tracheobronchitis Initiative to Conduct Antibiotic Evaluation*) para avaliar se uma estratégia de tratamento antibiótico de espera vigilante não é inferior ao tratamento antibiótico de rotina para traqueobronquite associada ao ventilador em relação aos dias sem ventilador mecânico.

**Métodos:**

O VATICAN é um estudo randomizado, controlado, aberto e multicêntrico de não inferioridade. Os pacientes com suspeita de traqueobronquite associada ao ventilador sem evidência de pneumonia associada ao ventilador ou instabilidade hemodinâmica devido a uma provável infecção serão designados para uma estratégia de espera vigilante, sem administração profilática de antimicrobianos contra traqueobronquite associada ao ventilador e prescrição de antimicrobianos somente em casos de pneumonia associada ao ventilador, sepse ou choque séptico, ou outro diagnóstico infeccioso, ou para uma estratégia de tratamento antimicrobiano de rotina por 7 dias. O desfecho primário será o número de dias sem ventilador mecânico em 28 dias, e um desfecho secundário importante será a sobrevida sem pneumonia associada ao ventilador. Por meio de uma estrutura de intenção de tratar com análise de sensibilidade por protocolo, a análise do desfecho primário abordará a não inferioridade com margem de 20%, o que se traduz em uma diferença de 1,5 dia sem ventilador. Outras análises seguirão uma estrutura de análise de superioridade.

**Conclusão:**

O VATICAN seguirá todos os padrões éticos nacionais e internacionais. O objetivo é publicar o estudo em um periódico geral de alta visibilidade e apresentá-lo em conferências de cuidados intensivos e doenças infecciosas para divulgação. Estes resultados provavelmente serão imediatamente aplicáveis à beira do leito após a conclusão do estudo e fornecerão informações com baixo risco de viés para o desenvolvimento de diretrizes.

## INTRODUÇÃO

O uso de ventilação mecânica invasiva (VMI) para suporte de órgãos é comum em unidades de terapia intensiva (UTIs), com prevalência estimada que varia de 36 a 89%.^(1-3)^ Apesar de ser um procedimento capaz de salvar vidas, a VMI está associada a complicações, como pneumonia associada ao ventilador (PAV) e traqueobronquite associada ao ventilador (TAV).^(4,5)^ Embora a incidência dessas complicações tenha diminuído nas últimas décadas devido a melhorias nas medidas preventivas e nos processos de cuidado,^(6)^ estima-se que a incidência de TAV ainda seja de 11% em pacientes que recebem VMI.^(7)^ Os pacientes com TAV têm períodos mais longos de ventilação mecânica, internações em UTI e internações hospitalares.^(7,8)^ As estimativas sugerem que aproximadamente 12% dos casos de TAV progridem para PAV,^(7)^ sugerindo que essas duas entidades podem estar em um *continuum*.^(9)^ Entretanto, não está clara a relação entre TAV e aumento da mortalidade.^(7)^

Embora muitos estudos avaliem estratégias para a prevenção da TAV,^(10)^ há dados limitados de ensaios clínicos para determinar se a TAV justifica o tratamento com antibióticos e apenas um ensaio clínico em andamento, que ainda não foi publicado.^(11)^ Dados observacionais mostraram um possível benefício do tratamento com antibióticos na redução da progressão da TAV para a PAV^(7,12)^ e um possível benefício na mortalidade,^(13)^ mas uma metanálise não mostrou benefício do tratamento com antibióticos nos desfechos clínicos.^(10)^ Além disso, as diretrizes da *Infectious Diseases Society of America* (IDSA)^(5)^ recomendam o não tratamento rotineiro da TAV com antibióticos (recomendação fraca, baixa qualidade de evidência). Pesquisa *on-line*, que incluiu 288 UTIs de 16 países,^(14)^ revelou heterogeneidade considerável no uso de antibióticos para o tratamento de TAV, sendo que 42% dos entrevistados relataram o uso de antibióticos em todos os pacientes com TAV, enquanto 26% não os usaram de forma alguma; o restante usou antimicrobianos apenas em circunstâncias específicas, como na presença de disfunção cardiovascular. Assim, observa-se aparente discrepância entre a prática clínica e as diretrizes atuais. Essas limitações das evidências atuais são reconhecidas pela heterogeneidade no tratamento da TAV na prática clínica e reforçam a necessidade de mais estudos na área.^(5,6,15-17)^

O estudo VATICAN (*Ventilator-Associated Tracheobronchitis Initiative to Conduct Antibiotic Evaluation*) foi concebido para abordar essa lacuna, avaliando se uma estratégia de tratamento antibiótico de espera vigilante não é inferior ao tratamento antibiótico de rotina na TAV em relação aos dias sem ventilador (DSVs) entre pacientes ventilados invasivamente com diagnóstico clínico de TAV. Nossa hipótese é a de que a estratégia de espera vigilante levará a um menor consumo de antimicrobianos, sem impacto na mortalidade ou na duração da ventilação mecânica, possivelmente às custas de um maior risco de PAV.

## MÉTODOS

Neste relato, usamos as recomendações para ensaios intervencionistas (SPIRIT).^(18)^ O relato final do ensaio seguirá a declaração *Consolidated Standards of Reporting Trials* (CONSORT) e sua extensão para ensaios de não inferioridade.^(19)^ O ensaio foi registrado no ClinicalTrials.gov (NCT05266066) antes da inclusão do primeiro paciente.

### Desenho

O VATICAN é um estudo clínico de não inferioridade, multicêntrico, randomizado, controlado, aberto, iniciado pelo investigador, conduzido para comparar se uma estratégia de tratamento antibiótico de espera vigilante é não inferior ao tratamento antibiótico por 7 dias para o tratamento de TAV.

O estudo será realizado em até 50 UTIs brasileiras. Ele está parcialmente inserido na IMPACTO MR,^(20)^ uma plataforma de pesquisa que coleta dados observacionais prospectivos de mais de 50 UTIs no Brasil, sendo que o VATICAN também permite a inclusão de UTIs fora da IMPACTO MR.

### População do estudo

A população-alvo do VATICAN são pacientes com suspeita de TAV que não apresentam choque séptico e nem evidência clínica de PAV. Os pacientes que forem ventilados mecanicamente por mais de 48 horas em cada local participante serão avaliados diariamente pelos pesquisadores do local para verificar a elegibilidade. Pacientes ventilados mecanicamente serão examinados durante as 3 primeiras semanas de ventilação mecânica, ou seja, não estarão sob ventilação mecânica prolongada. Nosso objetivo é incluir pacientes que atendam aos seguintes critérios clínicos para TAV: (1) tenham apresentado pelo menos um evento de leucocitose, leucopenia, desvio à esquerda, febre ou hipotermia e (2) atendam a pelo menos um critério de piora das secreções traqueais. Os principais critérios de exclusão foram pacientes com instabilidade hemodinâmica devido a uma provável infecção; alta suspeita de PAV (devido a um novo infiltrado pulmonar evidente ou piora persistente da troca gasosa); que estavam atualmente em uso de agentes antimicrobianos ou que tinham outra indicação de tratamento antimicrobiano. Esses critérios de exclusão garantem que nenhum paciente randomizado no estudo terá *Clinical Pulmonary Infection Score* (CPIS) ≥ 7, que foi demonstrado em estudo recente ser muito específico (0,89) e ter alto valor preditivo positivo (0,88) para o diagnóstico de PAV.^(21)^ A figura 1 apresenta a triagem do estudo e a avaliação de elegibilidade para o recrutamento no estudo. Os critérios detalhados de inclusão e exclusão estão descritos na tabela 1.

**Figura 1:** Triagem dos participantes do estudo e triagem do estudo até a randomização. ITRI - infecção do trato respiratório inferior; TC - tomografia computadorizada; TRI - trato respiratório inferior; PAV - pneumonia associada à ventilação mecânica.

**Tabela 1:** Critérios de inclusão e exclusão do estudo VATICAN

**Critérios de inclusão**
1. Pacientes adultos (idade ≥ 18 anos)
2. Admitidos na UTI
3. Ventilação mecânica invasiva por pelo menos 48 horas
4. Presença de critérios clínicos para PAV, definidos como: a. Temperatura > 38°C ou < 36°C **OU** leucócitos > 12.000/mL ou < 4.000/mL ou > 10% de formas imaturas **E** b. Novo aparecimento de secreções traqueais purulentas **OU** mudança nas características da secreção **OU** aumento no volume das secreções **OU** aumento da necessidade de aspiração
5. Amostra microbiológica do trato respiratório inferior em análise ou coleta no dia da triagem do estudo
6. Imagem do tórax (radiografia ou tomografia computadorizada do tórax) no dia da triagem do estudo
**Critérios de exclusão**
1. Gravidez ou lactação ativa
2. Uso atual ou indicação de uso de antibióticos sistêmicos por qualquer motivo
3. Instabilidade hemodinâmica, definida como hipotensão de início recente que não responde a bólus de fluido ou aumento da dose de vasopressor > 0,1mcg/kg/minuto de norepinefrina ou equivalente nas 6 horas anteriores
4. Piora na troca de gases, definida como um aumento na fração inspirada de oxigênio ≥ 20% ou aumento na PEEP ≥ 3cmH_2_O após período de estabilidade de ≥ 2 dias
5. Ventilação mecânica prolongada, definida como duração da ventilação mecânica invasiva ≥ 21 dias
6. Nova opacidade pulmonar na imagem do tórax, sugestiva de novos infiltrados infecciosos
7. Doença pulmonar prévia que prejudique a interpretação radiográfica para o diagnóstico de PAV
8. Diagnóstico prévio de PAV durante a hospitalização
9. Neutropenia (contagem absoluta de neutrófilos < 1.000/mL)
10. Imunossupressão grave conhecida
11. Traqueostomia no dia da triagem do estudo
12. Inclusão prévia no estudo nos últimos 30 dias
13. Previsão de retenção ou retirada de suporte de vida dentro de 7 dias
14. Pacientes sem probabilidade de sobreviver > 48 horas
15. Recusa de consentimento de participação no estudo

UTI - unidade de terapia intensiva; PAV - pneumonia associada ao ventilador; PEEP - pressão positiva expiratória final.

### Randomização, ocultação de alocação e cegamento

Os pacientes serão randomizados em uma proporção de 1:1 para cada braço de tratamento (Grupo Espera Vigilante *versus* Grupo Tratamento com Antibióticos por 7 dias). As listas de randomização serão geradas por um estatístico independente usando o software R^(22)^ em blocos de tamanhos aleatórios para preservar a ocultação da alocação e serão estratificadas por centro e por suspeita de diagnóstico de pneumonia viral. A randomização será realizada por meio de um sistema central de randomização *on-line* (RedCap), disponível 24 horas por dia.

Trata-se de estudo aberto, em que tanto os pacientes quanto os pesquisadores não estão cegados em relação às intervenções do estudo, dada a natureza dele e a necessidade de seguir os esquemas de tratamento locais conforme a microbiologia local. Os avaliadores e os estatísticos do estudo não terão conhecimento da atribuição do tratamento.

### Tratamentos do estudo

A figura 2 apresenta os principais elementos do protocolo do estudo. No Grupo Espera Vigilante, os participantes não receberão antimicrobianos para TAV. No entanto, os antimicrobianos podem ser usados, a critério do médico, quando houver outras indicações não relacionadas à TAV, como progressão para PAV, piora hemodinâmica ou choque, infecção altamente provável ou recém-confirmada pelo médico responsável pelo tratamento, disfunção orgânica inexplicável, nova ou piorada, ou qualquer outra indicação clínica validada para o uso de antimicrobianos. A iniciação antimicrobiana não é recomendada se as culturas do trato respiratório inferior forem positivas e o paciente não desenvolver outras indicações de tratamento.

**Figura 2:** Descrição do procedimento do estudo durante a janela do protocolo de tratamento de 7 dias. TAV - traqueobronquite associada ao ventilador; ITRI - infecção do trato respiratório inferior; PAV - pneumonia associada ao ventilador; SOFA - Sequential Organ Failure Assessment; TRI - trato respiratório inferior.

No Grupo Tratamento com Antibióticos, os participantes receberam antimicrobianos por 7 dias para o tratamento de TAV. Permitem-se 5 dias de tratamento para pacientes com melhora clínica completa, definida por ausência de febre, leucocitose e melhora nas secreções traqueais. Ajustes no tratamento antimicrobiano empírico serão feitos conforme os resultados microbiológicos estiverem disponíveis, se apropriado. A escolha do antimicrobiano será alinhada com a do antimicrobiano empírico recomendado para infecções do trato respiratório inferior adquiridas na UTI com base na microbiota de cada unidade. Fornecemos opções de antimicrobianos recomendadas pela diretriz em centros onde não se sugere um esquema empírico. Também serão permitidos antimicrobianos para indicações não relacionadas à TAV no Grupo Tratamento com Antibióticos.

Incentiva-se que cada UTI que inscrever pacientes no estudo siga as diretrizes de melhores práticas e seu protocolo institucional para o tratamento de pacientes críticos,^(23)^ incluindo um protocolo de prevenção de PAV. No entanto, o VATICAN não influenciará diretamente as melhores práticas em cada local. Não será permitido o uso de agentes antimicrobianos inalatórios durante o estudo. O Material Suplementar contém mais informações sobre os procedimentos do estudo.

### Desvios de protocolo

Como o estudo é aberto, podem ocorrer desvios de protocolo. Além disso, devido à sua natureza de não inferioridade e à necessidade de análises por protocolo, os desvios de protocolo serão acompanhados rigorosamente e avaliados pelo centro de coordenação, segundo critérios predefinidos.

No Grupo Tratamento com Antibióticos, os desvios de protocolo serão considerados se o paciente não receber antibióticos dentro de 24 horas após a randomização ou se receber menos de 7 dias de tratamento com antibióticos, salvo os pacientes aptos a receber tratamento de duração mais curta (pacientes com melhora clínica completa), que devem receber pelo menos 5 dias de tratamento com antibióticos.

No Grupo Espera Vigilante, o início do tratamento antimicrobiano após a randomização dentro da janela do protocolo de 7 dias de tratamento será considerado desvio do protocolo na ausência de outra indicação clara para o início do tratamento com antibióticos, conforme descrito.

### Desfechos do estudo

O desfecho primário é DSVs em 28 dias. Os DSVs são definidos como ausência de VMI por pelo menos 48 horas (sucesso na extubação).^(24)^ Se o paciente for reintubado dentro de 48 horas após a extubação, esse intervalo será tratado como zero DSVs. Se a reintubação for necessária após 48 horas, esse período será contado como DSVs. Os pacientes submetidos a uma traqueostomia serão considerados não dependentes de ventilação mecânica quando tolerarem nebulização contínua (com ou sem oxigênio), permitindo períodos curtos (< 1 hora/dia) de ventilação com pressão positiva. Os pacientes que receberem alta do hospital vivos antes de 28 dias serão considerados vivos e sem ventilação mecânica em 28 dias. Os não sobreviventes até 28 dias serão considerados como tendo zero DSVs.

Os desfechos secundários são: sobrevida sem PAV em 28 dias (desfecho secundário principal), mortalidade por todas as causas em 28 dias, incidência de PAV em 14 e 28 dias, número de dias fora da UTI em 28 dias, número de dias sem antibiótico em 28 dias, alteração no *Sequential Organ Failure Assessment* (SOFA) entre a randomização e 7 dias e incidência cumulativa de bactérias multirresistentes (MDR) isoladas microbiologicamente em 28 dias. Os desfechos secundários de segurança são a incidência de infecções hospitalares com cultura positiva em 28 dias e eventos adversos graves em 28 dias.

O desfecho de PAV será julgado por pelo menos dois juízes que não conhecem a intervenção do estudo e que têm experiência no tratamento de pacientes em estado crítico com ventilação mecânica. Se surgirem discrepâncias, elas serão resolvidas por consenso entre os dois juízes. Se não houver consenso, um terceiro avaliador será responsável por tomar a decisão final sobre o diagnóstico de PAV.

Informações detalhadas sobre os desfechos e as definições do estudo estão disponíveis no Material Suplementar.

### Coleta de dados

Os dados dos pacientes serão coletados por meio de um formulário de relato de caso *on-line* (e-CRF) usando o REDCap,^(25,26)^ desenvolvido pela equipe de tratamento de dados do Hospital Sírio-Libanês. O acesso aos e-CRFs será protegido por senha e terá o apoio da equipe de tratamento de dados do estudo.

Dados demográficos e basais, incluindo o *Simplified Acute Physiology Score* (SAPS) 3, dados laboratoriais, dados sobre variáveis fisiológicas, uso prévio de antibióticos e uso de esteroides na randomização, entre outros, foram coletados para todos os participantes. Dados diários sobre o uso de antibióticos, culturas microbiológicas e suporte ventilatório foram coletados por até 28 dias. Informações adicionais sobre a coleta de dados estão disponíveis no Material Suplementar.

### Monitoramento de dados

Um gerente de dados centralizado e específico realizará o monitoramento dos dados quanto à sua qualidade e integridade. Os dados de origem também serão rotineiramente auditados *on-line* ou no local, de acordo com um procedimento predefinido. Durante a coleta de dados, os campos foram explicitamente elaborados para permitir a coleta de dados de alta qualidade, usando intervalos e critérios de validação. Mais detalhes sobre o monitoramento de dados estão descritos no Material Suplementar.

### Eventos adversos

Este é um estudo em pacientes em estado crítico, e reconhecemos que, devido ao seu estado clínico, há alto risco inerente de morte e de várias anormalidades laboratoriais, anormalidades de sinais vitais e sintomas diretamente relacionados à gravidade da doença subjacente, bem como às práticas habituais de tratamento em UTIs. Portanto, essas anormalidades não serão necessariamente consideradas eventos adversos, a menos que haja suspeita ou confirmação de que estejam relacionadas às intervenções do estudo.^(27)^ Todos os eventos adversos graves potencialmente relacionados de forma causal à intervenção do estudo serão relatados e investigados de acordo com o guia de boas práticas clínicas.

### Tamanho da amostra

O cálculo do tamanho da amostra fundamentou-se na demonstração de não inferioridade da estratégia de espera vigilante em comparação com a estratégia de uso de antibióticos por 7 dias. Um tamanho de amostra de 295 pacientes por grupo (total de 590 pacientes), com um teste *t* unilateral para não inferioridade (com um nível de significância de 0,05), tem 80% de poder para rejeitar a hipótese nula de que o número de DSVs em 28 dias no Grupo Espera Vigilante é inferior ao número de DSVs em 28 dias no Grupo Tratamento com Antibióticos por 7 dias, com margem de 20% e coeficiente de variação de 1,38,^(7)^ considerando possível perda de seguimento ou desistências de 10%.

A escolha da margem de não inferioridade de 20% foi motivada pelo que foi considerado aceitável pelo Comitê Diretor, do ponto de vista clínico. Essa margem de não inferioridade de 20% se traduz em uma diferença de 1,5 DSV em 28 dias, considerando a média de DSVs de 7,69 dias e o desvio-padrão (10,67) em pacientes com TAV de uma publicação anterior.^(7)^

### Análise preliminar

Planejamos realizar análises preliminares após a inclusão de um terço (196 indivíduos) e dois terços (392 indivíduos) da amostra estimada, por meio de um Comitê de Monitoramento de Dados e Segurança independente. O objetivo dessa análise é interromper o estudo precocemente por motivos de segurança se forem observados resultados claramente superiores no Grupo Tratamento com Antibióticos em relação ao desfecho primário (DSVs), que será determinado usando o limite de Haybittle-Peto^(28)^ (p < 0,001) em cada análise preliminar, sem ajustes do valor de p para análises sequenciais de grupo. Não houve planejamento específico *a priori* para encerrar o estudo por futilidade ou eficácia.

### Análise estatística

A análise principal seguirá a estrutura de intenção de tratar, em que a hipótese nula é que o número de DSVs até 28 dias no Grupo Espera Vigilante é menor do que o número de DSVs no Grupo Tratamento com Antibióticos por 7 dias por uma margem de 20%. Se o limite superior do intervalo de confiança de 95% para inferioridade do Grupo Espera Vigilante for < 20%, rejeitaremos a hipótese nula de inferioridade. Para estimar o efeito do tratamento no desfecho primário, bem como nas análises preliminares, será usado um modelo estatístico apropriado ajustado para variáveis de estratificação para esse tipo de desfecho, conforme o comportamento da variável: pode ser usado um modelo aditivo generalizado para escala de localização e forma com distribuição beta inflada de zero ou testes não paramétricos com o cálculo de medidas de efeito e intervalos de confiança usando técnicas de reamostragem (*bootstrap*). O tamanho do efeito será estimado segundo o modelo mais bem ajustado aos dados, com intervalo de confiança de 95% (IC95%) unilateral para não inferioridade. Se a hipótese de não inferioridade for satisfeita, a superioridade do resultado primário será testada considerando IC95% usando um procedimento de teste hierárquico fechado. Uma análise de sensibilidade primária dessa análise de não inferioridade será por protocolo. Embora as análises por protocolo sejam geralmente a estrutura de análise primária recomendada para estudos de não inferioridade, a estrutura de análise de intenção de tratar pode levar a estimativas mais conservadoras em estudos antimicrobianos.^(29)^ Valor de p de 0,05 será considerado para indicar significância estatística na análise final.

Também realizamos análises de sensibilidade pré-planejadas do desfecho primário, incluindo apenas pacientes com TAV confirmado microbiologicamente, e analisamos os grupos de randomização como variável instrumental. As análises de subgrupo pré-planejadas incluirão o seguinte: pacientes com ou sem diagnóstico da doença pelo coronavírus 2019 (COVID-19) ou outra pneumonia viral, idade ≤ 60 ou > 60 anos, diagnóstico de trauma na admissão, lesão cerebral aguda, duração da VMI ≤ 5 ou > 5 dias no momento da randomização e tratamento antimicrobiano empírico eficaz *versus* ineficaz para patógenos isolados.

Nos desfechos que incluem a PAV como desfecho secundário, realizaremos análises de sensibilidade com base na definição de PAV, incluindo a definição mais específica e mais sensível de PAV (como casos possíveis e prováveis de PAV). Essas definições são apresentadas no Material Suplementar.

Todas as análises secundárias seguirão a estrutura usual de superioridade para análise. Mais detalhes sobre o modelo estatístico do desfecho primário, desfechos secundários, análises de sensibilidade e de subgrupo e apresentação dos dados serão descritos detalhadamente no plano de análise estatística a ser publicado antes do fechamento do banco de dados, seguindo as recomendações internacionais de relato de ensaios clínicos randomizados.

### Ética e divulgação

Os participantes não estão aptos a fornecer consentimento devido à sua condição clínica (uso de VMI). Portanto, o consentimento será obtido de seu parente mais próximo e/ou representante legal. Dada a tempestividade da intervenção, os participantes podem ser randomizados, e o consentimento do estudo pode ser obtido dentro de 48 horas após sua inclusão no estudo. O estudo foi elaborado de acordo com as diretrizes de boas práticas clínicas e segue os princípios do Documento das Américas, tendo sido aprovado pelo Comitê de Ética do Hospital Sírio-Libanês (CAAEE 48749421.0.1001.5461) e pelos Comitês de Ética em Pesquisa de todos os locais participantes.

A confidencialidade será garantida durante todo o processo, da coleta de dados ao tratamento de dados. Apenas serão compartilhados dados não identificáveis.

O estudo será enviado para publicação após a conclusão, independentemente de seus resultados. Nosso objetivo é publicar o estudo em um periódico geral de alta visibilidade e apresentá-lo em conferências sobre cuidados intensivos e doenças infecciosas para divulgação. Os dados gerados por este estudo serão disponibilizados mediante solicitação ao Comitê Diretor do estudo, com proposta de pesquisa cientificamente sólida.

## DISCUSSÃO

O VATICAN é um grande ensaio clínico multicêntrico e randomizado que avalia se se deve tratar a VAT na ausência de sinais de choque séptico ou de critérios de alto risco para PAV. Devido à sua natureza aberta, ele deve ser visto como um estudo de estratégias de tratamento com antibióticos, não do tratamento com antibióticos em si. É provável que esses resultados informem futuras diretrizes sobre essa condição.

Ao desenhar o VATICAN, os pesquisadores examinaram questões essenciais que merecem ser discutidas. A questão mais fundamental foi elaborar um estudo que aumentasse o recrutamento de centros em ambos os extremos da equipoise para esse estudo, ou seja, ambos os elementos para aqueles que se sentem fortes em relação ao tratamento rotineiro da TAV e aqueles que se sentem fortes em relação a não tratar a TAV, exceto em circunstâncias excepcionais.

O estudo foi inicialmente considerado de superioridade de eficácia do braço de tratamento de 7 dias, dadas as recomendações atuais das diretrizes para não tratar a TAV rotineiramente. No entanto, após a discussão no Comitê Diretor, o parecer obtido dos prováveis centros participantes e considerando as evidências publicadas atualmente de que o tratamento é comumente introduzido, decidimos que o teste de hipótese de não inferioridade teria maior probabilidade de fornecer evidências além de uma dúvida razoável, especialmente para os médicos que têm forte opinião sobre o tratamento da TAV. Evidências robustas são essenciais se um estudo clínico tiver como objetivo final mudar a prática clínica. Como a hipótese de não inferioridade levou a um tamanho de amostra maior, se qualquer um dos braços do estudo tiver desempenho melhor do que os outros, uma análise de eficácia pode ser realizada usando abordagens frequentistas padrão e taxas de erro tipo I aceitáveis.

O cegamento e a escolha do agente antimicrobiano em cada local também são preocupações. Considerando a microbiologia variável das infecções do trato respiratório inferior nas UTIs brasileiras,^(30)^ era improvável que uma única estratégia de tratamento antimicrobiano fosse apropriada para todos os locais participantes. Além disso, essa tática não representaria a prática clínica dos locais e, em última análise, prejudicaria a generalização do estudo. Portanto, decidimos que a melhor estratégia de projeto seria um projeto não cego, no qual a escolha do agente antimicrobiano é deixada a cargo do local do estudo, de acordo com os padrões locais de resistência antimicrobiana. Essa estratégia apresenta novos desafios na implementação do estudo, mas aumenta a aceitação da intervenção e o recrutamento de centros para o estudo. Por outro lado, a forma como os antimicrobianos são prescritos não será monitorada devido à natureza pragmática da aplicação da intervenção, embora isso possa ser uma possível limitação.

O recrutamento de participantes foi outra preocupação durante o desenho do estudo. Dados brasileiros não publicados sugerem que a maioria dos pacientes de UTI com ventilação invasiva é submetida a tratamento antimicrobiano durante um longo período de internação. Ao recrutar locais, reforçamos o compromisso de cada local com o uso racional de antimicrobianos, o que pode ser visto como uma consequência positiva não intencional do estudo. À medida que o recrutamento se desenvolve, essa é uma parte crítica planejada das visitas aos locais do estudo para otimizar o recrutamento.

Outra armadilha do estudo é a adesão às intervenções, dada a sua natureza não mascarada e o quão fortemente os clínicos individuais podem se sentir em relação à terapia antimicrobiana, em que os clínicos geralmente são parciais em relação ao uso da terapia antimicrobiana mais do que o necessário por vários motivos.^(31)^ Essa questão será monitorada especificamente durante a implementação do estudo, avaliando a separação entre os grupos de tratamento, conforme o tratamento com antibióticos durante os primeiros 7 dias após a randomização. No intuito de reduzir os possíveis vieses na análise final, reforçamos a adjudicação dos desvios de protocolo com critérios claros, como os aqui descritos. A duração do tratamento é outra escolha de desenho do nosso estudo. Escolhemos 7 dias de tratamento no braço tratamento de rotina para permitir a separação fisiológica do Grupo Espera Vigilante, porém não consideramos um desvio de protocolo se o tratamento for reduzido para 5 dias em pacientes com melhora clínica completa. Foram propostos períodos de tratamento mais curtos (3 dias), mas esses não são atualmente a prática padrão nas UTIs brasileiras.

Por fim, a escolha do desfecho primário foi outra questão importante no desenho do estudo. Embora a incidência de PAV possa ser vista como desfecho interessante para o VATICAN, estudos recentes que avaliaram a PAV como desfecho primário não demonstraram benefícios em outros desfechos clínicos quando a PAV é evitada,^(32,33)^ o que sugere que a PAV pode ser melhor vista como um desfecho substituto que não está necessariamente relacionado a outros desfechos clínicos. Portanto, escolhemos DSVs como nosso desfecho primário, o que equilibraria os benefícios e danos da intervenção em um único desfecho composto, mais centrado no paciente. Além disso, outros desfechos secundários, como dias sem antibióticos e dias fora da UTI, fornecerão elementos adicionais para a avaliação dos benefícios e danos dos esquemas de tratamento.

### Perspectiva

Quando redigimos o esboço deste protocolo de estudo, mais de 180 pacientes foram incluídos no estudo, e 32 centros foram treinados e recrutados desde junho de 2022. Com até 50 centros ativos, esperamos que o estudo seja concluído até 2026. No entanto, as armadilhas e barreiras ao recrutamento descritas podem acarretar taxas de recrutamento mais lentas, que serão abordadas pelo Comitê de Coordenação do estudo.

## CONCLUSÃO

O VATICAN representa um esforço considerável para avaliar uma questão de pesquisa de longa data que ainda não foi adequadamente abordada por estudos randomizados multicêntricos. É provável que os resultados sejam imediatamente aplicáveis à beira do leito após a conclusão do estudo e forneçam informações com baixo risco de viés para o desenvolvimento de diretrizes.
